# Induction of Human iPSC-Derived Cardiomyocyte Proliferation Revealed by Combinatorial Screening in High Density Microbioreactor Arrays

**DOI:** 10.1038/srep24637

**Published:** 2016-04-21

**Authors:** Drew M. Titmarsh, Nick R. Glass, Richard J. Mills, Alejandro Hidalgo, Ernst J. Wolvetang, Enzo R. Porrello, James E. Hudson, Justin J. Cooper-White

**Affiliations:** 1 Australian Institute for Bioengineering & Nanotechnology, The University of Queensland, St. Lucia, QLD 4072, Australia; 2 School of Biomedical Sciences, The University of Queensland, St. Lucia, QLD 4072, Australia; 3 School of Chemical Engineering, The University of Queensland, St. Lucia, QLD 4072, Australia; 4 Biomedical Manufacturing, Manufacturing Flagship, CSIRO, Clayton, Victoria 3169, Australia

## Abstract

Inducing cardiomyocyte proliferation in post-mitotic adult heart tissue is attracting significant attention as a therapeutic strategy to regenerate the heart after injury. Model animal screens have identified several candidate signalling pathways, however, it remains unclear as to what extent these pathways can be exploited, either individually or in combination, in the human system. The advent of human cardiac cells from directed differentiation of human pluripotent stem cells (hPSCs) now provides the ability to interrogate human cardiac biology *in vitro*, but it remains difficult with existing culture formats to simply and rapidly elucidate signalling pathway penetrance and interplay. To facilitate high-throughput combinatorial screening of candidate biologicals or factors driving relevant molecular pathways, we developed a high-density microbioreactor array (HDMA) – a microfluidic cell culture array containing 8100 culture chambers. We used HDMAs to combinatorially screen Wnt, Hedgehog, IGF and FGF pathway agonists. The Wnt activator CHIR99021 was identified as the most potent molecular inducer of human cardiomyocyte proliferation, inducing cell cycle activity marked by Ki67, and an increase in cardiomyocyte numbers compared to controls. The combination of human cardiomyocytes with the HDMA provides a versatile and rapid tool for stratifying combinations of factors for heart regeneration.

Human pluripotent stem cells (hPSCs)^1^,^2^ and their differentiated progeny are highly applicable as human cellular models for cell types where primary human cells are difficult to obtain, for example, contractile cardiomyocytes^3^. Additionally, the indefinite self-renewal of hPSCs combined with highly efficient directed differentiation protocols^4^ facilitates the large-scale production of specific human cell types. Human cell-based assays utilising such differentiated progeny are thus gradually replacing animal cell equivalents, and have clear relevance in many different applications including developmental biology, therapeutic discovery, personalised medicine, drug development, and regenerative medicine^5^.

A central pursuit for researchers pursuing cardiac regeneration is the induction and control of proliferation in differentiated cardiomyocytes, particularly adult cardiomyocytes, which otherwise exhibit very low levels of proliferation^6^. Since the neonatal mouse heart has been shown to transiently retain regenerative potential^7^, strategies to reactivate proliferation in damaged adult heart have attracted interest for therapeutic application^8^,^9^. The mechanisms driving post-natal cardiomyocyte cell cycle arrest in mammals are still poorly understood and so there is great potential in identifying factors that effectively modulate cardiomyocyte proliferation and stimulate cardiomyocytes to re-enter the cell cycle and divide.

To this end, several screening studies have been reported in zebrafish, mouse, and rat systems, revealing novel regulators of cardiomyocyte proliferation (comprehensively reviewed in reference^10^). Zebrafish studies, using a genetically encoded cell cycle reporter (FUCCI), screened kinase inhibitors to identify Hedgehog (Hh), insulin-like growth factor (IGF) and transforming growth factor-β (TGF- β) as being involved in cardiomyocyte proliferation^11^. Integrin and PI3K signalling has also been identified by studies in adult rat ventricular myocytes^12^, and the IGF/PI3K/AKT pathway was also implicated in proliferation using hPSC-derived cardiomyocytes^13^. Various other studies have resulted in identification of a multitude of stimuli as being potentially important for cardiomyocyte proliferation, including neuregulin^14^, FGF-1 and inhibition of p38 MAP kinase^15^, G-CSF^16^, Hippo^17^, and Wnt^18^. A screen of mouse PSC-derived cardiomyocytes also showed that GSK3β inhibition, p38 inhibition and an inducer of ERK phosphorylation promoted proliferation^19^. Although several of these identified pathways are also known to be active in development/differentiation of cardiomyocytes^3^, they appear to have a proliferative effect on cells that are terminally differentiated and functional. Because of the number of candidate pathways and the potential for cross-talk, it would be advantageous to screen multiple factors in combination to rate their maximal proliferative response and look for preferential combinations. However, as yet very few systematic screens for cardiomyocyte proliferative agents have been conducted using human cardiomyocyte populations. The availability of large numbers of functional human cardiomyocytes through recent progress in cardiac differentiation of hPSCs^3^ now means that human cardiomyocytes can be accessed in abundant quantities for diverse *in vitro* experimental investigations in high throughput. Comparative *in vitro*/*in vivo* studies of cardiomyocyte proliferation have indeed revealed relevance to *in vivo* predictivity^16^, suggesting this may be a rewarding approach to identifying drugs for cardiac regeneration.

Whilst hPSC-derived cardiomyocytes are now accessible, platforms that provide miniaturisation, parallelisation and high-throughput assessment are still emerging, the advantages of which having been highlighted previously^20^,^21^,^22^,^23^,^24^,^25^,^26^,^27^. At the molecular biology scale, microfluidic valves and reaction chambers have previously been integrated at high density^28^ in the thousand-plex order, and commercialised for applications in genomics by Fluidigm Corporation^29^. On the cellular scale, an array of ~1600, 4.1 nL microfluidic cell culture chambers for single cell isolation and culture has been successfully used to track clonal HSC proliferation^30^. In addition, a myriad of assay operations can be achieved with microfluidics, including single cell culture with genetic profiling^24^ and manipulation^31^; embryoid body screening^25^; extracellular matrix screening^26^; logarithmically^21^, spatiotemporally^32^, or gradient-based^33^ varied soluble factor concentrations; and even mechanical stimulation^34^. Microscale technologies have also previously been exploited for cardiomyocyte enrichment^35^, patterning^36^, and mechanical^37^ and electrical characterisation^38^.

In this work, we increased the throughput of miniaturised cell cultures on a single microfluidic chip by developing a High Density Microbioreactor Array (HDMA) platform. This microfluidic platform is an 8100-plex cell culture array, with a 20 nL culture volume and 0.2 mm^2^ culture area, accommodating cell populations of tens to hundreds of cells at relevant surface densities used in adherent mammalian cell cultures (10^4^–10^5 ^cells/cm^2^). The HDMA is an evolution of our previously reported microbioreactor array platform^39^,^40^,^41^. Importantly, in this embodiment, the HDMA is now capable of generating a full-factorial/combinatorial array of four exogenous factors, combined with a substantial increase in the number of serially-connected culture chambers, to potentially allow increased, differential levels of autocrine/paracrine factor accumulation, and also parallel replicate culture chambers of each condition to improve measurement accuracy in a single array. The HDMA detailed in this paper incorporates 2 parallel replicates, 50 serial chambers, 3 factor concentration levels and 4 factors to give 2 × 50 × 3^4^ = 8100 total cell culture chambers on a single microfluidic chip.

The HDMA platform thus enables two essential functions: firstly, the ability to access miniaturised cultures of representative cell populations, at a high level of integration, which is vital for high-throughput experimentation; and secondly, the ability to autonomously generate a detailed spectrum of culture environments from a selection of input media. This in turn streamlines the combinatorial investigation of a set of factors of interest, which is particularly relevant to probing signalling pathways that induce cardiomyocyte proliferation. In this work, we present and validate the HDMA platform, confirming its capability to culture hPSCs and thereafter employ it in a combinatorial screen of putative proliferation factors in hPSC-derived cardiomyocytes.

## Results

### HDMA Platform Design and Fabrication

HDMAs were based on a similar design architecture to that used previously^39^,^41^, and were designed using the same layout algorithms. The new design developments were the expansion to four factors, extension to 50 serial culture chambers, parallel replication of device columns, and downsizing of the culture chamber dimensions (from 1.63 to 0.513 mm diameter). The HDMA’s microfluidic architecture can accommodate an array of culture chambers comprising *a* parallel replicates, *b* serial replicates, *c* factor concentration levels and *d* factors resulting in *n* = *abc*^*d*^ total array elements. This throughput scales favourably with physical complexity, requiring only 2*c* fluidic inputs, no integrated valves, and only two polymer device layers on a substrate layer, whilst achieving valveless control of fluid multiplexing, metering and routing to a large number of arrayed culture chamber elements. This represents a 30-fold (~1.5 log) increase in array elements that are incorporated on-chip, compared to the previous embodiment of this architecture^39^,^40^,^41^.

**Figure 1:**
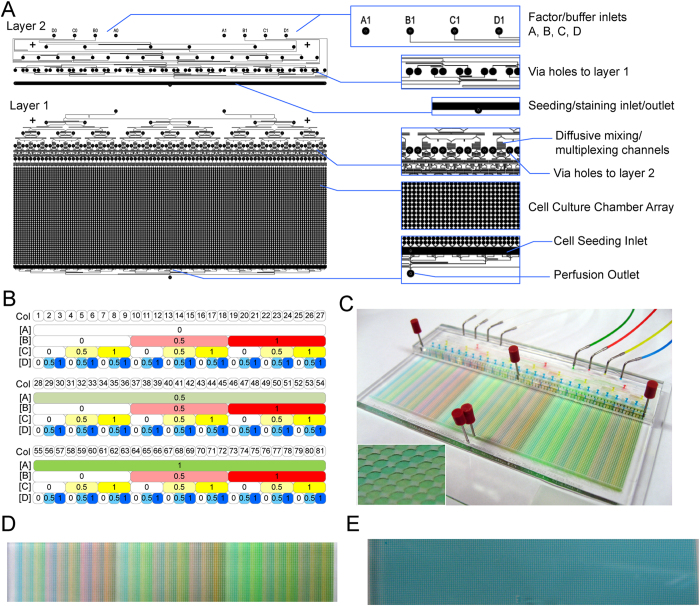
HDMA Design and Validation. (**A**) HDMA photomask design with key features marked. (**B**) Matrix of design normalised concentration levels for factors A–D, in each of the 81 column-pairs in the array. (**C**) Photograph of assembled device filled with green, red, yellow, and blue food dye solutions representing factors A–D, and PBS representing buffers A–D. Inset – magnified detail of cell culture chambers. (**D**) Photograph of cell culture array section as in Panel (**C**), showing detail of generated conditions. (**E**) Photograph of cell culture array showing complete fluid replacement after injection of blue dye from perfusion outlet.

The HDMA design and key elements incorporated are shown in Fig. 1A, and the main physical parameters of the HDMA platform under nominal flow conditions are given in Supplementary Table 1. The spectrum of conditions the HDMA is designed to generate from four generic factors A, B, C, and D is shown in Fig. 1B, and comprises 81 distinct compositions of the three concentration levels for each of the four factors. Each of these compositions is supplied to two replicate columns that run parallel to each other in the culture chamber array, such that the array contains 81 column-pairs (Fig. 1A). HDMA features that were microfabricated with dry film photoresist were reproducible between individual wafers, at 106.9 ± 2.37 μm (S.D., n = 5 wafers), and were uniform across a single wafer, at 107.3 ± 5.71 μm (S.D., n = 5 points measured). Feature sizes designed to 50 μm (the smallest features on chip) showed excellent fidelity of feature definition (Supplementary Figure 1).

### HDMA Performance Validation

HDMA performance was validated by dye loading of green, red, yellow and blue food dye solutions as factors and PBS solutions as buffers. This confirmed dye partitioning into the correct columns of the cell culture array, and multiplexing of 81 distinct dye combinations from four stock solutions and four buffers (Fig. 1C,D). Injection of a single blue dye solution at the device outlet was then able to completely replace fluid in the cell culture chambers – a necessary function during device coating, cell seeding, and immunostaining (Fig. 1E).

**Figure 2:**
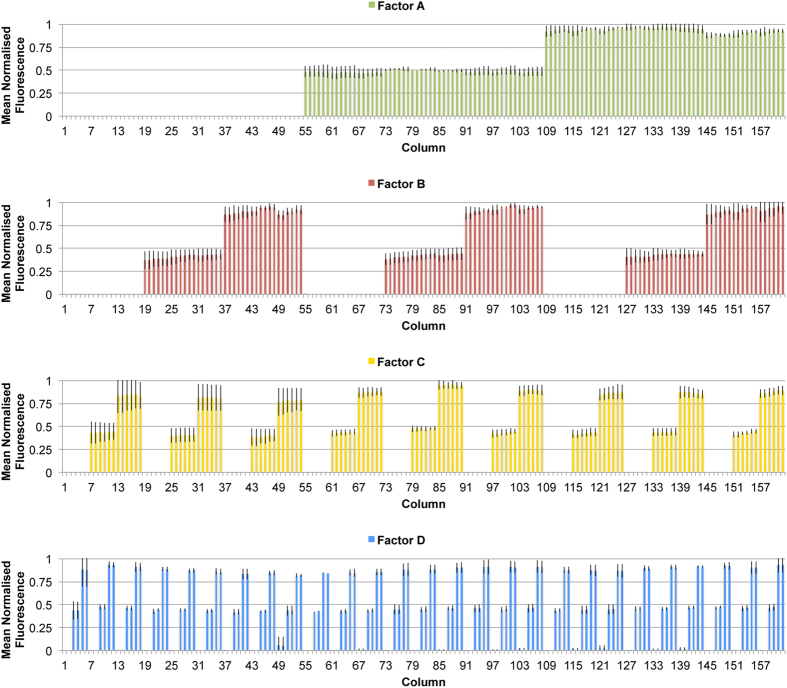
HDMA Concentration Level Validation. Fluorescence quantification in each of the 162 columns (81 column-pairs) in the array (corresponding to matrix in Fig. 1B), using 40 kDa FITC-dextran dye and quantitative fluorescence microscopy. Bars represent mean ± S.D. of n = 3 independent experiments including separately fabricated HDMA devices, and fluorescence is normalised to the blank (0) and maximum (1) fluorescence for that device/channel.

Concentration levels in each of the 162 columns in the HDMA, corresponding to 81 column-pairs with 81 distinct soluble factor compositions (Fig. 1B), were quantified with 40 kDa FITC-dextran dye and fluorescence microscopy. This confirmed that the diffusive mixing and multiplexing fluidics were accurately metering and routing the designed compositions of factors to their respective columns (Fig. 2). This also confirmed that molecules up to 40 kDa in size could be adequately mixed by the device at flowrates up to 400 μL/h total flow, and implicitly showed that fluid flow was evenly distributed between columns. The compositions generated by HDMAs were reproducible between independently fabricated devices (Fig. 2).

### Cell Seeding and Attachment in HDMAs

**Figure 3:**
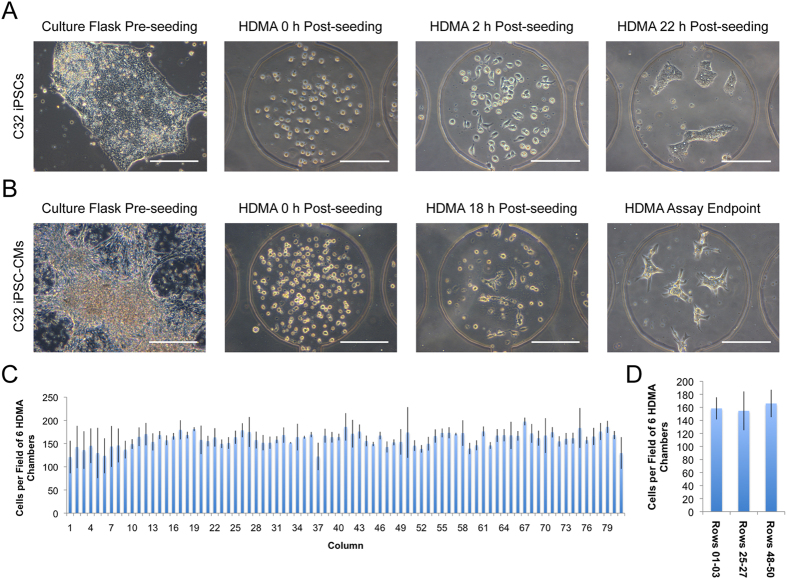
Cell seeding and attachment in HDMAs. (**A**) Phase contrast micrographs of C32 iPSCs prior to and following seeding into HDMA. Scale bars: 200 μm. (**B**) Phase contrast micrographs of C32 iPSC-CMs from 15 d cardiac differentiations, prior to and following HDMA seeding. Scale bars: 200 μm. (**C,D**) Plots of the distribution of fixed, Hoechst-labelled HES3 hESCs after injection into HDMA at 2 × 10^6 ^cells/mL. Image fields containing 6 HDMA chambers (three rows of one column pair) were acquired and nuclei counted by image cytometry. Images fields were taken from the top (Rows 01-03), middle (Rows 25–27), and bottom (Rows 48–50) section of the device, and from each of the 81 column-pairs. Average numbers of cells detected per field of 6 chambers is shown, averaged along column pair (**C**; n = 3 row groups) and row group (**D**; n = 81 column pairs). Bars represent mean ± S.D. and data from one experiment is shown to highlight the variation present within an individual HDMA.

We investigated the seeding and attachment of hPSCs and hPSC-derived CMs in HDMAs, both to confirm that they could be accommodated as a starting point for cell-based assays, and to evaluate the uniformity of cell distribution throughout the HDMA. Injection of live hPSCs showed initiation of attachment within 2 h of seeding and without exchange of culture medium, and by 22 h cells had readily attached and formed several small colonies per culture chamber (Fig. 3A). Likewise, day 15 hPSC-derived cardiomyocyte populations were tested. Attachment appeared less rapid than for hPSCs, as at 18 h post-seeding there was a mixture of single attached cells, grouped attached cells, live cells in the early stage of attachment, and dead cells and debris. However, cells continued to attach and spread over 2–3 days with static feeding every 24 h, and remained attached as characteristic clusters of beating cells with phase-bright borders (Fig. 3B and Supplementary Videos 1&2).

To measure the distribution of cells across the columns of the HDMA, we injected fixed, Hoechst-labelled cells (HES3 hESCs), and performed quantification of nuclei by image cytometry. Image fields containing 6 HDMA chambers (3 rows from the two columns in a column pair) were taken for each of the 81 column pairs across the device and from the top (Rows 01–03), middle (Rows 25–27) and bottom (Rows 48–50) sections of the device. This revealed reasonably uniform distribution of cells across the whole HDMA cell culture array (Supplementary Figure 3). The coefficient of variation of the number of cells in each image field was 14.8%, which is higher than the ~10% we have observed previously for hESC seeding in the previous generation of this bioreactor with larger culture chambers^39^. The impact of this variance may however be overcome by the greater replication of culture chambers available in this generation. The distribution of cells in fields across the 81 column-pairs in the device was uniform, as it was between the top, middle and bottom rows of a column (Fig. 3C,D). The outermost individual columns in the device were observed to have a slightly lower than average cell density (Supplementary Figure 2).

### High Throughput Cell-Based Assays in HDMAs: hPSC-Derived Cardiomyocyte Proliferation Screen

**Figure 4:**
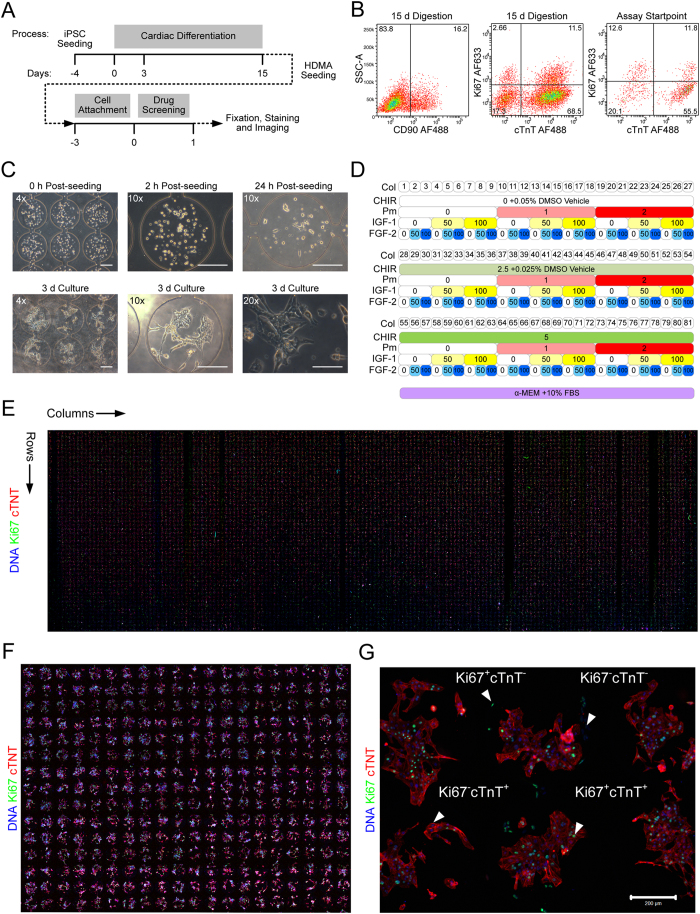
hPSC-derived Cardiomyocyte Proliferation Screening in the HDMA Platform. (**A**) Experimental timeline used in the screen. (**B**) Representative flow cytometric analyses of cell population used in HDMA screening, with percentages marked. Dot plots of CD90^+^ stromal cell content from 15 d digestion of differentiated hPSCs; dot plots of Ki67 and cTnT expression in both 15 d digestion of differentiated hPSCs and cells replated for 2 d prior to assay startpoint. (**C**) Representative phase contrast micrographs of CMs before and after seeding, and cultured for up to 3 d with drug treatments, as indicated. Scale bars for indicated objective magnification: 4×, 200 μm; 10×, 200 μm; 20×, 100 μm. (**D**) Matrix of design culture conditions in each of the 81 column pairs in the array, for the screened factors: CHIR99021 (CHIR), μM; Purmorphamine (Pm), μM; IGF-1, ng/mL; FGF-2, ng/mL. DMSO was included so as to be at a uniform total concentration of 0.05% v/v in the CHIR channels, acting as a vehicle control. (**E**) Tile-scan confocal image of the entire HDMA (~91 × 28 mm) treated for 24 h and used in further analysis, after fixation and *in situ* immunostaining for Ki67, cTnT and DNA. (**F**) Higher-magnification confocal image of subsection of replicate HDMA treated for 3 d, showing cell population arrangement in arrayed culture chambers. (**G**) Confocal image showing detail of individual HDMA chambers from replicate HDMA treated for 3 d, demonstrating presence of various proliferating (Ki67^+^) and non-proliferating (Ki67^−^) myocyte (cTnT^+^) and nonmyocyte (cTnT^−^) populations. Scale bar: 200 μm.

We next demonstrated cell–based assays in the HDMA with hPSC-derived cell types of interest, in this case hPSC-derived cardiomyocyte populations. Here, we tested the capacity of the HDMA to screen putative proliferation-inducing factors, following the scheme given in Fig. 4A. The input population typically contained ~80% cardiac troponin T (cTnT)^+^ cardiomyocytes, and ~20% cTnT^−^ non-myocytes (mostly CD90^+^ stromal cells) (Fig. 4B), and contained a high proportion of spontaneously contractile cells prior to dissociation. Typically, 10–15% of cells at this stage were positive for Ki67 (Fig. 4B).

Cardiomyocyte populations were seeded into HDMA chambers to attach (Fig. 4C). 24 h after seeding, cardiomyocytes had begun to attach, with some spontaneously contracting. Cells were allowed 3 d in total to attach (with daily medium exchanges), prior to assay with the panel of factors. After 3 d attachment, the majority of cells present in each chamber were attached and spontaneously contracting (Supplementary Video 1). Cardiomyocytes could be cultured for an additional 3 d in the HDMA while maintaining typical morphology and spontaneous contractions (Fig. 4C). The panel of factors we screened consisted of: CHIR99021 (CHIR), a small molecule GSK3β inhibitor/canonical Wnt activator^42^; Purmorphamine (Pm), a small molecule Hedgehog pathway activator^43^; insulin-like growth factor 1 (IGF-1); and fibroblast growth factor 2 (FGF-2). The combinatorial panel generated by the HDMA based on these factors (Fig. 4D) was applied under continuous flow for 24 h. At the endpoint, cells had appeared to respond differentially under the various treatments: some conditions appeared to have undergone proliferation and had stopped beating, whereas others were still contractile (Supplementary Video 2).

*In situ* immunodetection of the cell nuclei, proliferation marker Ki67 and cardiomyocyte marker cTnT in the screened cardiomyocyte populations was then used as an endpoint readout to differentially stain proliferating (Ki67^+^) and non-proliferating (Ki67^−^) myocyte (cTnT^+^) and nonmyocyte (cTnT^−^) populations. Confocal tile-scan imaging of the entire HDMA chip revealed differential prevalence of the various populations across the 81 column-pairs in the HDMA (Fig. 4E). Cell culture was successful across the overwhelming majority of the 8100 cell culture chambers, although infrequently we detected some columns that were devoid of cells or not stained properly due to blockages caused by cell aggregates in the microfluidic channels during cell seeding or growth. Overall, the HDMA contained an 8100-plex array of miniaturised cell cultures that were neatly delineated and of uniform general appearance at the multi-chamber level (Fig. 4F). The HDMA contained rich information on differential population contents within individual chambers (Fig. 4G).

### High Content Screening in HDMAs

**Figure 5:**
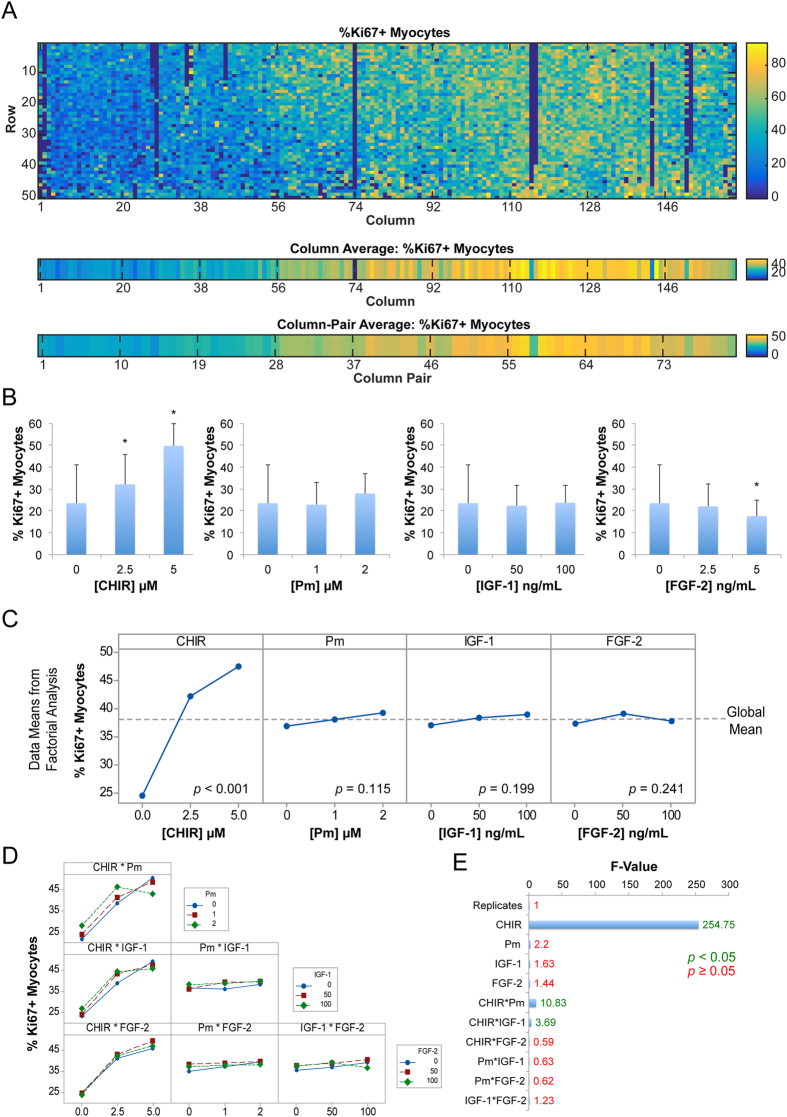
High-Content Screening and Factorial Analysis of Myocyte Proliferation in HDMA. (**A**) Top panel: Heatmap showing percentages of Ki67^+^ proliferating cardiomyocytes in each of the 8100 chambers in the HDMA. Lower panels: Heatmaps of mean values of %Ki67^+^ myocytes in each column and column-pair in the HDMA. (**B**) Data from individual column-pairs representing treatment with individual factors in HDMA. Bars represent mean and error bars represent S.D. of 50 rows from each column-pair where corresponding chambers in the 2 replicate columns are averaged. *indicates *p* < 0.05 versus control (no factors) by unpaired two-tailed *t*-test. (**C**) Plots of individual factor effects as calculated by factorial analysis. The global mean across all HDMA conditions is shown. *p*-values for factor effect significance shown are calculated by MINITAB for each factor. (**D**) Plots of interaction effects for combinations of two factors. Concentrations are as in Panel (**C**). (**E**) Plot of F-value from factorial analysis showing variation explained by individual factors and combinations of 2 factors, with F-value marked for each. Green represents *p* < 0.05, red represents *p* ≥ 0.05.

To extract absolute numbers of proliferating cardiomyocytes from every chamber in the HDMA, we performed high content screening on images of individual chambers from the HDMA using cell segmentation and analysis software (CellProfiler; see Supplementary Figure 2), coupled to image/flow cytometry software (Flowing Software or FCS Express Plus). We measured the percentage of cTnT^+^ myocytes that were also Ki67^+^ after 24 h of treatment with the various factor combinations, as the primary response variable. The cardiomyocyte population was firstly gated based on the cTnT^+^ cellular projected area and cellular integrated intensity of cTnT staining. Then, Ki67^+^ cells were identified based on the integrated intensities of nuclear Ki67 staining. The percentage of proliferating myocytes (Ki67^+^cTnT^+^) was then mapped for every chamber in the HDMA (Fig. 5A).

Taking data from individual HDMA column-pairs that contained each dose of each of the individual factors, it was clear that FGF-2, IGF-1 and Pm failed to significantly increase the percentage of proliferating myocytes, with 100 ng/mL FGF-2 slightly decreasing it (Fig. 5B). Clear increases in percentages of Ki67^+^ myocytes were apparent with 2.5 or 5 μM CHIR treatment, up to twice the percentage of untreated cells (Fig. 5B).

As evidence for the delivery of the protein growth factors, FGF-2 and IGF-1, which are presumably more labile than the small molecules, a weak proliferative response to FGF-2 by the non-myocyte population was detected in the HDMA (Supplementary Figure 4A), so this acts as an on-chip positive control showing the proteins are delivered. We have also previously demonstrated delivery of labile proteins including FGF-2, TGF-β1, Activin A, BMP-4 over longer periods in the similar MBA system^39^,^40^. The proliferative effect was also weak in the static controls (Supplementary Figure 4B), with approximately the same magnitude, suggesting there is no substantial difference due to, for example, adsorption between the two culture formats.

This method of analysis, considering only individual column-pairs with specific treatments, provides a direct test of the effect of each treatment, but does not make use of the plethora of data across the whole HDMA. On the other hand, factorial analysis, a design-of-experiments statistical technique, uses data from all conditions across the HDMA where a factor is present in estimating average factor effect magnitudes. This approach better exploits the data available through large-scale integration of parallel experimental conditions in the combinatorial array of conditions in the HDMA. Therefore, we performed factorial analysis to better estimate the effects of both individual factors and combinations of factors.

Similar to data from individual column-pairs, factorial analysis using all the columns in the HDMA to estimate factor effects showed a large effect size for CHIR, with the percentage of Ki67^+^ myocytes increasing strongly with increasing CHIR concentration. When these individual factor effects were mapped relative to the global mean for all conditions in the HDMA, CHIR was shown to have a strongly positive effect, which was statistically significant (Fig. 5C). In this plot, lines that track the global mean show that changing the concentration of the factor has no effect, and for Pm, IGF-1 and FGF-2 the effect of their addition was flat, with the percentage of Ki67^+^ myocytes essentially insensitive to their dosage. Their factor effects were also deemed to be insignificant. This analysis shows that relative to the global mean across all HDMA chambers, the absence of CHIR was strongly negative, 2.5 or 5 μM CHIR was strongly positive, and the presence or absence of the remaining factors showed no appreciable bias away from the global mean (Fig. 5C).

According to factorial analysis, combinations of any other individual factors with 5 μM CHIR did not result in further increases in levels of proliferating myocytes, however supplementing 2.5 μM CHIR with either 2 μM Pm or 100 ng/mL IGF-2 was able to increase the response seen with 2.5 μM CHIR alone to a level more comparable with 5 μM CHIR (Fig. 5D), suggesting perhaps these factors could substitute for insufficient dosage of CHIR. Although the factorial analysis marked these interactions as significant (Fig. 5E), it should be noted that it is unclear whether this represents a genuine positive interaction because the effect sizes were small and made up only a small proportion of the variation compared to CHIR’s effect. In an analysis of the variance explained by each factor, or combinations of two factors together, CHIR was by far the most dominant source of variance in the factorial analysis, as shown by a comparison of F-values (Fig. 5E).

The analysis, both by direct titration in individual column-pairs, and factorial analysis of multiple factor conditions across the entire HDMA chip, therefore points to CHIR as a potent inducer of human cardiomyocyte proliferation.

### Confirmation of Effects of HDMA Screening Hits in Standard Static Culture

**Figure 6:**
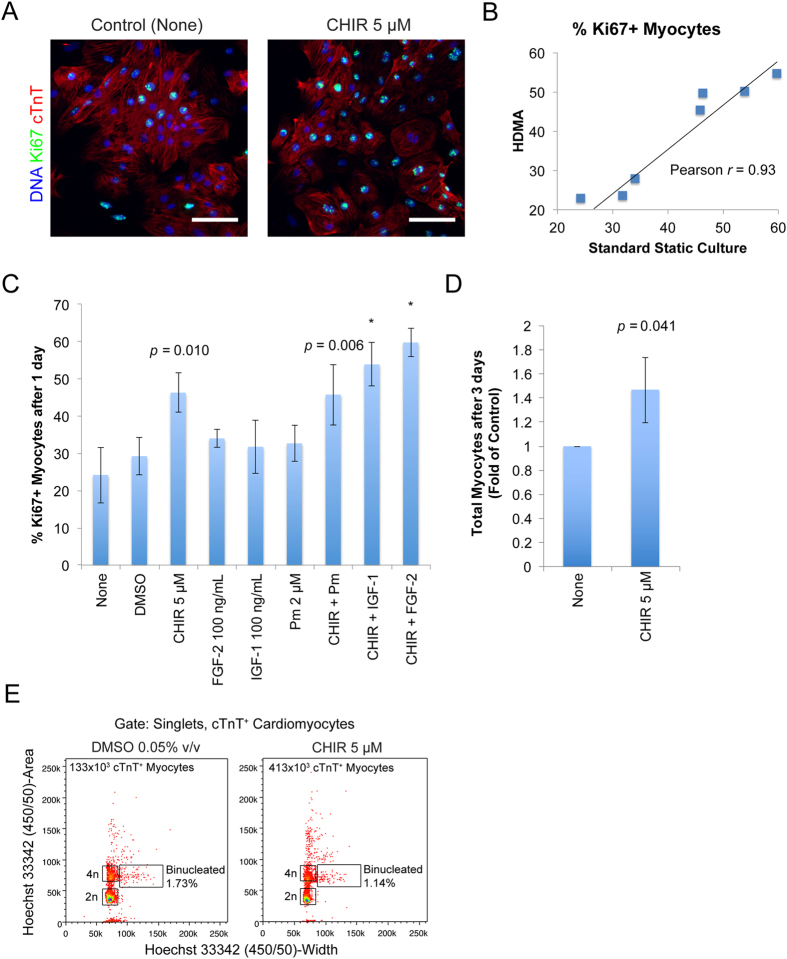
Confirmation of HDMA Screen Results in Standard Static Cultures. (**A**) Confocal fluorescence images of Ki67 expression in myocyte population after 24 h treatment with Control medium or medium containing 5 μM CHIR. Scale bar: 100 μm. (**B**) Scatterplot of results from HDMA individual column-pairs against conditions tested in standard static culture, with linear regression and correlation coefficient marked. (**C**) Quantification of percentage Ki67^+^ myocytes after 24 h treatment in static cultures. Bars represent mean ± S.D. of 2–3 independent experiments including separate cardiomyocyte inductions. *indicates *p* < 0.05 by one-way ANOVA with Tukey post-hoc tests vs. None condition. No other differences were significant. *p*-values (paired two-tailed *t*-test) against None condition are indicated for treatments with large effect magnitudes. (**D**) Quantification of total myocytes after treatment for 3 days with Control (None) medium or medium containing 5 μM CHIR. Bars represent mean ± S.D. of 4 independent experiments including inductions, normalised to control (None) condition. *p*-value (paired two-tailed *t*-test) indicated. (**E**) Quantification of percentage of binucleated cardiomyocytes after treatment for 3 days with Control (DMSO 0.05% v/v) medium or medium containing 5 μM CHIR. Dotplot from flow cytometric analysis of nuclear staining (Hoechst 33342) pulsewidth versus pulse area. Gating is on singlets by FSC and SSC area, height and width, and cTnT^+^ cardiomyocytes. Total number of cTnT^+^ myocytes estimated from cell counting and percentage cTnT^+^ content is marked.

To confirm the efficacy of CHIR as a proliferation-inducing agent, and the measured effects of the other factors identified in the HDMA screen, follow-up experiments were carried out using standard static culture plates. Day 15 cardiomyocyte preparations were plated for 3 d, similar to HDMAs. These populations contained both cTnT^+^ myocytes and cTnT^−^ non-myocytes at the assay start point. After 24 h of treatment in static culture, cells treated with CHIR (5 μM) had a clearly increased percentage of Ki67 expression in cTnT^+^ myocytes relative to controls (Fig. 6A).

Quantification of Ki67+ myocytes in standard static culture reflected the factor effects seen in the HDMA (Fig. 6B), with a strong Pearson’s correlation coefficient of *r* = 0.93, meaning the HDMA delivered data that were highly concordant with standard static culture-based assays, despite being conducted in a highly miniaturised and parallelised format. Addition of 5 μM CHIR increased the percentage of proliferating myocytes on average from 24% to 46%, similar to the ~2-fold effect seen in the HDMA (Fig. 6C). Although this was not statistically significant at the 5% significance level by ANOVA, this represents a robust effect magnitude when considered over 3 separate inductions of cardiomyocytes, which generate populations with varying baseline levels of proliferation and non-myocyte content. Direct comparison of this condition against the control revealed a significant *p*-value of 0.010 (paired two-tailed *t*-test) (Fig. 6C). Pm, IGF-1 or FGF-2 did not elicit significant increases above the baseline, much the same as HDMA results. Combinations of these factors with CHIR performed similarly to CHIR alone, and did not result in further statistically significant increases above that which CHIR could achieve alone (Fig. 6C). CHIR’s effect when combined with IGF-1 or FGF-2 was more robust in that it was statistically different to control conditions, compared to when applied alone (Fig. 6C). Importantly, when treated with CHIR at 5 μM for an extended period of 3 days, the total number of cTnT^+^ cardiomyocytes detected was significantly increased over control cells (*p* = 0.041) (Fig. 6D). To preclude the possibility that CHIR treatment induces karyokinesis without cytokinesis, resulting in binucleated cardiomyocytes, we performed flow cytometric analysis of static control cultures (Fig. 6E). This revealed that the proportion of binucleated cells (detected by pulsewidth of nuclei staining, gated on singlet cardiomyocytes) was both low (~1%), and not appreciably different between 3 day DMSO- versus CHIR-treated cultures, yet CHIR treated cultures had substantially more cTnT+ cardiomyocytes, indicative of *bona fide* induction of cardiomyocyte proliferation and cytokinesis, without increases in binucleation. This suggests that CHIR induces cardiomyocyte proliferation marked by Ki67^+^ nuclei followed by cytokinesis, resulting in elevated numbers of cTnT^+^ myocytes. The effects of CHIR seemed to be more pronounced in the myocyte fraction, as measurements of proliferating non-myocytes (Ki67^+^cTnT) showed a small effect in the HDMA (Supplementary Figure 4A), but this was not significant when measured in static controls (Supplementary Figure 4B), suggesting CHIR’s effect was targeted towards cardiomyocyte rather than non-myocyte proliferation.

## Discussion

In developing the HDMA, we attempted to maximise the experimental capacity (range of conditions surveyed, output data throughput, and technical accuracy of measurement) relative to the effort expended in conducting an experiment. Thus, we capitalised on the massive parallelisation capacity of microfabrication techniques to achieve multiplexed fluidic metering and routing to a maximal number of culture elements, whilst minimising peripheral connections and manual handling operations. We were able to incorporate 8100 cell culture chambers by maximising integration towards practical constraints set by the maximum size of our photolithography and soft lithography processing, which is based on 6-inch silicon wafer masters, and 100 × 76 mm slides as a device substrate, and the minimum size of individual array elements. To our knowledge, this work represents one of the largest scale integrations of parallel cell culture chambers on a single microfluidic chip thus far.

The size of a chamber needs to accommodate a sufficient number of cells for the relevant cell type or assay, a number that is determined in part by the population heterogeneity and the number of cells required to make a statistically relevant measurement. On this note, analysis of single cells is important for deconstructing population heterogeneity. While this system could be utilised for single-cell culture, either by exploiting the cell seeding distribution among the 8100 chambers or by including cell traps, single cell culture is not necessarily relevant to cell processing at larger scale. In this case, we thought it useful to obtain data on single cells, but in their normal culture context (i.e. culture in colonies or non-colony monolayers) that might be employed in cell-based assays or cell bioprocessing. We therefore coupled the high-throughput array of culture conditions in the HDMA with image cytometry^44^ as an analysis method. Image cytometry allows for both absolute cell number enumeration, and for a rich set of phenotypic features to be extracted *in situ* from individual cells within a population of tens to hundreds of cells in each chamber of the HDMA. This increases the data yield from a single HDMA experiment considerably, and provides great versatility in assay readouts. The HDMA constitutes a highly versatile platform for cell-based assays, expands experimental parameter space with high-throughput generation of culture conditions, is compatible with hPSCs and hPSC-derived cell types, and can generate detailed datasets at single-cell resolution using high content screening tools.

Microfluidic miniaturisation and parallelisation also leads to a significant reduction in reagent requirements, compared to an equivalent experiment in a standard microplate format, although this can be difficult to explicitly define. We conservatively estimate a minimum 10-fold reduction in culture and analysis reagent cost, compared to a minimally equivalent experiment in 96-well plates. By reducing the experiment layout onto a single microfluidic chip rather than multiple multi-well plates, the HDMA also reduces the associated manual fluid handling requirements and sample handling overhead for labelling cells with analytical reagents and imaging, and significantly reduces imaging area, which can be a bottleneck in high-content screens. For population-level analyses from HDMAs, we find that low resolution, wide-field or tile-scanning imaging perform well, and others have suggested faster acquisition using DNA microarray scanners^45^, to which some assays might be readily adaptable. Single-cell-level analyses that utilise image cytometry generally need a minimum resolution such that cell nuclei are more than several pixels in diameter, for accurate segmentation and measurements of feature intensity and shape parameters. This was readily achievable with standard imaging systems using the HDMA.

HDMAs proved compatible with the necessary functions of a cell-based assay: seeding and attachment of cells, treatment of cells with stimuli, and endpoint analysis of the cell populations. The HDMA performed impressively in demonstration of a complex cell-based assay using a mixed population of hPSC-derived cardiomyocytes and stromal cells. Using image cytometry, we were able to provide absolute enumeration and phenotypic information for every cell in every chamber, measuring cTnT^+^ cardiomyocytes while excluding the non-myocyte fraction from analysis, as well as extracting Ki67 expression status. We were able to measure increases in hPSC-derived cardiomyocyte proliferation in response to treatment with candidate inducer molecules. Importantly, this was done in 8100 parallelised culture chambers with a high rate of success across the array, and with extremely strong correlation to standard static culture outcomes (Pearson’s correlation coefficient of *r* = 0.93, Fig. 6B). Results from both the individual column-pair analysis (comparing the direct titration of each factor) agreed with the array-wide comparison of factor effects through factorial analysis. This highlights the effectiveness of the HDMA in miniaturising and improving the throughput of cell-based assays whilst maintaining a high degree of fidelity and accuracy.

hPSC-derived cardiomyocytes are immature and typically display a decreasing level of proliferation over time in culture, until reaching a minimal level of proliferation when more matured^46^. Cardiomyocytes derived in RPMI-based, chemically defined protocols also show baseline proliferation^4^. The propensity of the cells to respond to proliferative signals may be dependent on the timing of their differentiation, which tends to vary between individual inductions for individual hPSC lines, and can be a source of variation to consider in repeat experiments. Nevertheless, the HDMA screen identified the appropriate conditions inducing proliferation that were reflected in static controls conducted over several independent inductions, confirming its utility in high throughput screening of factors regardless of the subtleties of the differentiation state of the cell line in question. Although we derived the cardiomyocytes with a serum-free, RPMI-based protocol, α-MEM containing FBS was used as the background medium used for screening. This could potentially mask the effects of more subtle inducers of cardiomyocyte proliferation, however we elected to use it with the day 15 cTnT^+^ differentiated cells to prioritise the health of the cardiomyocyte population during dissociation, replating, attachment and survival in the HDMA and static controls. This was to preclude the possibility that supplemented factors were simply enhancing the health of the culture (and thereby baseline proliferation) rather than inducing proliferation above baseline levels. Even in the presence of FBS, we identified strong proliferative responses after 1 day of treatment in our screen.

The continuously perfused culture environment in the HDMA permits insights that cannot be obtained from static plates. Of note, some differences in responses between the HDMA and static plates were observed and this could reflect a possible role for paracrine factors in cardiomyocyte proliferation. In the HDMA, continuous flow of medium means cells towards the top of a column see “fresh” medium, whereas cells at the bottom see a more “conditioned” medium that has had exogenous factors depleted, and has a higher accumulation of endogenous secreted factors. In this work, we saw that CHIR directly induced cardiomyocyte proliferation from the first rows of the HDMA. This effect was clear across virtually all CHIR-treated column-pairs (column-pairs 28–81; Fig. 5A). When present at 2.5 μM, there was some reduction in proliferation activity towards the lower rows of the HDMA, but at 5 μM the effect was more sustained, penetrating through all rows at a comparable level (Fig. 5A). This may suggest slight depletion and insufficiency of CHIR at the 2.5 μM level in the most downstream rows, relative to 5 μM CHIR. Alternatively, negative feedback loops mediated by extracellular regulators of Wnt signalling may be involved. Although we averaged responses along columns in the HDMA and this faithfully reflected bulk responses in standard static culture, further experimentation and analysis with the HDMA platform might be used to unravel endogenous factor contributions, by targeting for example extracellular regulators of the Wnt pathway.

The HDMA screen and subsequent follow-up experiments in static culture plates provided evidence for hPSC-derived cardiomyocyte proliferation in response to stimulation with the GSK3β inhibitor CHIR99021. This is in line with observations made by other studies across multiple species using another widely used but less specific GSK3β inhibitor, BIO. In neonatal rat cardiomyocytes, BIO was shown to induce cell cycle progression and cell division, as well as cell cycle progression in adult rat cardiomyocytes^18^. In mouse pluripotent stem cell-derived cardiomyocytes, both BIO and CHIR were shown to induce proliferation^19^. BIO was also shown to be effective in hPSC-derived cardiomyocytes^19^. Context for the effectiveness of these GSK3β inhibitors is provided by the various roles of GSK3β in the heart^47^.

Contrary to previous reports using zebrafish or rodent models, we did not identify in the HDMA screen any significant role for any of the other factors (the Hedgehog agonist Purmorphamine, IGF-1, or FGF-2), either alone or in combination (i.e. no additive or synergistic impacts), in inducing cardiomyocyte proliferation. When these factors were provided in combination with CHIR, there was also no effect above that which CHIR alone could achieve, save for the apparent substitutive effect of Pm or IGF-1 when CHIR was provided at a sub-optimal level of 2.5 μM (Fig. 5D). This was reflected in standard static culture controls, as these combined treatments were not significantly different to CHIR alone (Fig. 6C). This may suggest that, even if these factors were involved in proliferation, there may be a convergence on the GSK3β pathway as a final effector of myocyte proliferation, in line with recent reports^17^,^48^. Since temporal modulation of Wnt signalling is effective in differentiating hPSCs to cardiomyocytes, and this requires initial activation of Wnt to induce early mesoderm formation followed by inhibition of Wnt to specify cardiac cells^49^, it is interesting to note that re-activation of Wnt signalling with CHIR can then be used to promote proliferation of the cardiomyocyte-specified cells. Future characterisation of the gene expression signatures and functional aspects of CHIR-expanded cardiomyocytes will add to our understanding of this process. In addition, the use of cardiomyocytes matured by further growth factor exposure or electro/mechanically-stimulated culture processes would allow us to assess whether mature, post-mitotic cardiomyocytes are still sensitive to induction of proliferation. In summary, these studies identify potentially important species differences with regards to induction of cardiomyocyte proliferation and highlight the importance of screening factor impacts using human cardiomyocytes. Furthermore, the ability to personalise these screens using patient-derived PSCs offers tremendous opportunities for patient-specific cardiac regenerative screens for personal drug stratification in the future.

## Methods

All fine chemicals were obtained from Sigma Aldrich and all cell culture and detection reagents were obtained from Life Technologies unless otherwise mentioned.

### HDMA Design and Fabrication

HDMA designs were produced in DraftSight software (Dassault Systemes), and printed at high resolution onto 7-inch HY2 photoplates (Konica Minolta) with a photoplotter (MIVA). HDMAs were fabricated to 100 μm feature height by photolithography and then replica molding. 6-inch silicon wafer substrates were cleaned (acetone, isopropanol, N_2_), after which two 50 μm-thick layers of PerMX 3050 dry film photoresist (DuPont) were laminated and processed according to the manufacturer’s directions. Feature heights were measured with an optical surface profilometer (Veeco NT1100) (Supplementary Figure 1). Standard replica molding with poly(dimethylsiloxane) (PDMS; Sylgard 184, Dow Corning)^50^ was then used to form PDMS casts of the master features. Via holes and inlet/outlet holes were punched in the PDMS layers using a biopsy punch. The two cast PDMS layers and a 100 × 76 mm glass microscopy slide (Proscitech) were then manually aligned and assembled using O_2_ plasma bonding.

### HDMA Dye Loading Tests and Fluorescence Quantification

For qualitative assessment of factor mixing, green, red, yellow and blue food dye solutions in PBS (representing factors A, B, C, D as in Fig. 1A), and PBS buffers (representing buffers A, B, C, D) were perfused into the HDMA at 400 μL/h total flowrate.

For quantitative validation of concentration levels in individual columns, 40 kDa FITC-dextran (100 μM solution in PBS) was perfused into one of the factor channels, with all other inlets containing PBS buffer, at 400 μL/h total flowrate. For quantification, chambers were imaged by an automated, inverted fluorescent microscope (Olympus IX81 cell^R system) using FITC filters, with exposure time chosen so as to give a linear response (*R*^2^ > 0.97) in integrated intensity with respect to solution concentration. Background signal from a blank chamber was subtracted from raw intensities, technical replicate chambers on chip were averaged, and intensities in individual factor channels were normalised to the minimum (0) and maximum intensity (1) in that channel.

### Cell Culture and Microplate Controls

Human pluripotent stem cell lines were dealt with under approval of the UQ Human Research Ethics Committee (approval numbers 2012000556 and 2012000557). HES3 human embryonic stem cells were obtained from Stem Cells Ltd. The WT2-iPS C32 (hereafter C32) episomally generated iPSC line was generated and characterised as described previously^51^. C32 hPSCs were adapted to passaging in single-cell suspension with TrypLE Express, and were maintained in mTeSR-1 medium (StemCell Technologies) on hESC-qualified Matrigel (BD Biosciences). Microplate controls were seeded with equivalent surface densities of cells as HDMAs. All media were supplemented with 1% v/v penicillin/streptomycin for experimentation, and 0.5% v/v penicillin/streptomycin for general cell maintenance.

### Cell Seeding Distribution Quantification

To evaluate the cell seeding distribution within the HDMA, HES3 hPSCs were detached with TrypLE Express treatment, and triturated with a micropipette. These were fixed with 4% paraformaldehyde/PBS, washed with PBS, stained with Hoechst 33342, and injected into HDMAs at 2 × 10^6 ^cells/mL. The HDMAs were imaged with an automated, inverted fluorescent microscope, and nuclei present in each column were quantified by image cytometry (see below).

### Validation of hPSC Attachment and Culture Initiation

HDMAs were autoclaved, dried in an oven, and then vacuum-filled with sterile PBS. For hPSC seeding, HDMAs were surface coated for two hours at room temperature with a single 1 mL injection of hESC-qualified Matrigel (BD Biosciences) at the manufacturer’s recommended concentration. C32 hPSCs maintained in mTeSR-1 cultures on Matrigel were detached with TrypLE Express and resuspended in mTeSR-1 medium containing 10 uM ROCK inhibitor Y-27632, then seeded into HDMAs at 4 × 10^6 ^cells/mL (4 × 10^4 ^cells/cm^2 ^surface density). A volume of approximately 800 μL of cell suspension was injected into HDMAs, which were then left in an incubator for cells to attach and initiate growth in colonies. During this period, mTeSR-1 medium without ROCK inhibitor was exchanged every 6 hours by a syringe pump.

### hPSC Cardiac Differentiation and Validation of Attachment and Culture Initiation within HDMA

Cardiomyocyte populations were derived from hPSCs using a protocol based on that described previously^52^. Briefly, C32 iPSCs were seeded at 4 × 10^4 ^cells/cm^2^ and cultured in mTeSR-1 on Matrigel for 4 days. Subsequently, they were exposed for 3 d to RPMI B27 medium (RPMI1640 + 2% v/v B27 supplement) containing BMP-4, Activin A, FGF-2 (all RnD Systems) and CHIR99021 (Stemgent) with daily medium exchange, then RPMI B27 medium containing IWP-4 (Stemgent) for 10 days and then RPMI B27 with medium exchange every 2–3 days. Beating sheets of cardiomyocytes were digested at 15 d with 0.2% collagenase type I (Sigma) in 20% fetal bovine serum in PBS (with Ca^2+^ and Mg^2+^) and subsequently 0.25% trypsin-EDTA. The cells were then filtered using a 100 μm mesh cell strainer (BD Biosciences) before seeding for experiments.

For iPSC-derived cardiomyocyte (iPSC-CM) seeding, HDMAs were surface coated for 1 h at room temperature with a single 1 mL injection of 0.1% gelatin solution, then rinsed with PBS. Digested C32 iPSC-CMs from day 15 differentiations (as described above) were resuspended in alpha-MEM + 10% FBS + 200 μM ascorbate-2-phosphate medium at a concentration of 10^7 ^cells/mL (for a target surface density 10^5 ^cells/cm^2^). A volume of approximately 400 μL of cell suspension was injected into HDMAs, which were placed in an incubator. During this period, medium was exchanged every 24 hours by a syringe pump. Feeding did not detach a significant proportion of cells.

### HDMA Cell-Based Assay: hPSC-Derived Cardiomyocyte Proliferation Screen

HDMAs were autoclaved, dried in an oven, vacuum-filled with sterile PBS, then coated with a 1 mL injection of 0.1% gelatin solution for 1 h, RT. Digested hPSC-derived cardiomyocytes were resuspended in α-MEM + 10% FBS + 200 μM ascorbate-2-phosphate medium, and seeded into HDMAs at 1 × 10^7 ^cells/mL (1 × 10^5 ^cells/cm^2^). Cells were incubated for 2–3 d (as indicated) to allow for their gradual attachment, and were fed daily during this time with an exchange of medium by syringe pump. Putative proliferation agents were then applied: CHIR99021 (Stemgent), Purmorphamine (Stemgent), IGF-1 (R&D Systems), and FGF-2 (Millipore). Owing to the design of the microfluidic mixing channels, these factors are supplied at 4× the highest concentration required to be screened, to allow for subsequent diffusive mixing and dilution. DMSO was included at 0.05% v/v (final concentration) in the buffer channel for CHIR99021, as a vehicle control. The final design compositions of each column are shown in Fig. 4D. Finally, continuous factor perfusion was initiated and continued at 24 μL/h total flow for 3 days.

### HDMA In-situ Immunostaining

After attachment and 3 days of subsequent factor treatment under continuous flow, HDMAs were analysed for endpoint markers. All solutions were perfused at 1 mL/h using a syringe pump for at least 300 μL total volume delivery. HDMAs were rinsed with a small volume of PBS then fixed with 1% paraformaldehyde/PBS solution (30 min, on ice), and rinsed with PBS. HDMAs were blocked/permeabilised with PBS containing 5% FBS and 0.2% Triton X-100 (30 min, RT), then primary antibodies (proliferation marker Ki67, 1:200 dilution, Cat#9129S, Cell Signaling Technology; cardiac troponin T, 1:200 dilution, Cat#MS295P1, Thermo Fisher Scientific) were applied in blocking buffer and left overnight at 4 °C. HDMAs were then washed with blocking buffer, then secondary antibodies (Alexa Fluor 488, Alexa Fluor 568; both 1:400) and Hoechst 33342 were applied in blocking buffer for 1 h at RT. HDMAs were finally rinsed with blocking buffer then PBS. Fixation did not grossly affect the cell morphology or remove cells. Microplate controls were stained in a similar fashion.

### Imaging, Image Cytometry and Data Processing

HDMAs were imaged by a Zeiss LSM 710 confocal microscope system (Carl Zeiss), incorporating tile-scanning of the entire cell culture array (approximately 91 × 28 mm) at 16-bit image depth, and in one optical section of ~250 um thickness. Fluorescence channels were acquired individually to minimise spectral overlap. The resulting montage image was sliced into images of individual chambers for image cytometry processing.

Image cytometry processing was performed with CellProfiler software (version 2.0, http://cellprofiler.org)^53^,^54^. Briefly, cell nuclei were segmented, cytoplasmic regions were identified by propagation from the cell nuclei, then nuclear and cytoplasmic size, shape, and intensity features were measured (see also Supplementary Figure 2). The resulting measurements were exported in a format readable by FCS Express Plus software (version 4, DeNovo Software, http://www.denovosoftware.com), according to the developer instructions (http://www.denovosoftware.com/site/manual/index.html?cell_profiler_export.htm), or for Flowing Software (http://www.flowingsoftware.com), cell feature data were merged with nuclear feature data, and spurious data from flagged chambers where image processing failed were replaced with zero measurements, using a custom MATLAB script. FCS Express Plus, Flowing Software, Microsoft Excel, and MATLAB software were then used for data processing and visualisation. MINITAB 15 software was used to perform factorial analyses, as described previously^39^, with interaction terms up to second order included in the model. GraphPad Prism software or Microsoft Excel were used for additional statistical testing, as described for each dataset.

### Flow Cytometry

The composition of the input population and microplate controls for the cardiomyocyte proliferation screen were analysed by flow cytometry for expression of cardiac troponin T (cardiomyocytes; MA5-12960, Thermo Fisher Scientific, 1:200), Ki67 (active cell cycle; mAb9129, Cell Signalling Technology, 1:200), and CD90 (stromal cells; MAB2067, RnD Systems, 2 μg/ml). Cells were fixed using 4% PFA for 15 min at room temperature. The cells were incubated in 5% FBS in PBS for CD90 or 5% FBS, 0.2% Triton-X-100 in PBS for cTnT/Ki67 for 45 min. They were then rinsed and incubated with secondary antibodies and Hoechst for 30 min at 4 °C. Cells were then run on a BD LSRII (BD Biosystems) and analyzed using WEASEL v2.7.4 (http://www.frankbattye.com.au).

## Additional Information

**How to cite this article**: Titmarsh, D. M. *et al*. Induction of Human iPSC-Derived Cardiomyocyte Proliferation Revealed by Combinatorial Screening in High Density Microbioreactor Arrays. *Sci. Rep.* **6**, 24637; doi: 10.1038/srep24637 (2016).

## Supplementary Material

Supplementary Information

Supplementary Video 1

Supplementary Video 2

## References

[b1] Takahashi K. . Induction of Pluripotent Stem Cells from Adult Human Fibroblasts by Defined Factors. Cell 131, 861–872 (2007). 18035408 10.1016/j.cell.2007.11.019

[b2] Thomson J. A. . Embryonic Stem Cell Lines Derived from Human Blastocysts. Science 282, 1145–1147 (1998). 9804556 10.1126/science.282.5391.1145

[b3] Burridge Paul W., Keller G., Gold, Joseph D. & Wu Joseph C. Production of De Novo Cardiomyocytes: Human Pluripotent Stem Cell Differentiation and Direct Reprogramming. Cell Stem Cell 10, 16–28 (2012). 22226352 10.1016/j.stem.2011.12.013 PMC3255078

[b4] Burridge P. W. . Chemically defined generation of human cardiomyocytes. Nat. Methods 11, 855–860 (2014). 24930130 10.1038/nmeth.2999 PMC4169698

[b5] Harris K. . Comparison of Electrophysiological Data From Human-Induced Pluripotent Stem Cell–Derived Cardiomyocytes to Functional Preclinical Safety Assays. Toxicol. Sci. 134, 412–426 (2013). 23690542 10.1093/toxsci/kft113

[b6] Pasumarthi K. B. S. & Field L. J. Cardiomyocyte Cell Cycle Regulation. Circ. Res. 90, 1044–1054 (2002). 12039793 10.1161/01.res.0000020201.44772.67

[b7] Porrello E. R. . Transient Regenerative Potential of the Neonatal Mouse Heart. Science 331, 1078–1080 (2011). 21350179 10.1126/science.1200708 PMC3099478

[b8] Senyo S. E., Lee R. T. & Kühn B. Cardiac regeneration based on mechanisms of cardiomyocyte proliferation and differentiation. Stem Cell Res. 13, 532–541 (2014). 25306390 10.1016/j.scr.2014.09.003 PMC4435693

[b9] Rodgers K., Papinska A. & Mordwinkin N. Regulatory aspects of small molecule drugs for heart regeneration. Adv. Drug Del. Rev. 96, 245–252 (2016). 10.1016/j.addr.2015.06.013 26150343

[b10] Sharma A., Zhang Y. & Wu S. M. Harnessing the Induction of Cardiomyocyte Proliferation for Cardiac Regenerative Medicine. Curr. Treat. Options Cardiovasc. Med. 17, 1–12 (2015). 10.1007/s11936-015-0404-z PMC4956404 26324824

[b11] Choi W.-Y. . *In vivo* monitoring of cardiomyocyte proliferation to identify chemical modifiers of heart regeneration. Development 140, 660–666 (2013). 23293297 10.1242/dev.088526 PMC3561784

[b12] Kühn B. . Periostin induces proliferation of differentiated cardiomyocytes and promotes cardiac repair. Nat. Med. 13, 962–969 (2007). 17632525 10.1038/nm1619

[b13] McDevitt T. C., Laflamme M. A. & Murry C. E. Proliferation of cardiomyocytes derived from human embryonic stem cells is mediated via the IGF/PI 3-kinase/Akt signaling pathway. J. Mol. Cell. Cardiol. 39, 865–873 (2005). 16242146 10.1016/j.yjmcc.2005.09.007 PMC3505759

[b14] Bersell K., Arab S., Haring B. & Kühn B. Neuregulin1/ErbB4 Signaling Induces Cardiomyocyte Proliferation and Repair of Heart Injury. Cell 138, 257–270 (2009). 19632177 10.1016/j.cell.2009.04.060

[b15] Engel F. B., Hsieh P. C. H., Lee R. T. & Keating M. T. FGF1/p38 MAP kinase inhibitor therapy induces cardiomyocyte mitosis, reduces scarring, and rescues function after myocardial infarction. Proc. Natl. Acad. Sci. USA 103, 15546–15551 (2006). 17032753 10.1073/pnas.0607382103 PMC1622860

[b16] Shimoji K. . G-CSF Promotes the Proliferation of Developing Cardiomyocytes *In Vivo* and in Derivation from ESCs and iPSCs. Cell Stem Cell 6, 227–237 (2010). 20207226 10.1016/j.stem.2010.01.002

[b17] Heallen T. . Hippo pathway inhibits Wnt signaling to restrain cardiomyocyte proliferation and heart size. Science 332, 458–461 (2011). 21512031 10.1126/science.1199010 PMC3133743

[b18] Tseng A.-S., Engel F. B. & Keating, Mark T. The GSK-3 Inhibitor BIO Promotes Proliferation in Mammalian Cardiomyocytes. Chem. Biol. 13, 957–963 (2006). 16984885 10.1016/j.chembiol.2006.08.004

[b19] Uosaki H. . Identification of Chemicals Inducing Cardiomyocyte Proliferation in Developmental Stage–Specific Manner With Pluripotent Stem Cells. Circ. Cardiovasc. Genet. 6, 624–633 (2013). 24141057 10.1161/CIRCGENETICS.113.000330 PMC3898889

[b20] Titmarsh D. M., Chen H., Wolvetang E. J. & Cooper-White J. J. Arrayed cellular environments for stem cells and regenerative medicine. Biotechnol. J. 8, 167–179 (2013). 22890848 10.1002/biot.201200149

[b21] Kim L., Vahey M. D., Lee H.-Y. & Voldman J. Microfluidic arays for logarithmically perfused embryonic stem cell culture. Lab Chip 6, 394–406 (2006). 16511623 10.1039/b511718f

[b22] King K. R. . A high-throughput microfluidic real-time gene expression living cell array. Lab Chip 7, 77–85 (2006). 17180208 10.1039/b612516f PMC3205973

[b23] Figallo E. . Micro-bioreactor array for controlling cellular microenvironments. Lab Chip 7, 710–719 (2007). 17538712 10.1039/b700063d

[b24] Kamei K.-i. . An integrated microfluidic culture device for quantitative analysis of human embryonic stem cells. Lab Chip 9, 555–563 (2009). 19190791 10.1039/b809105f

[b25] Kim C. . 3-Dimensional cell culture for on-chip differentiation of stem cells in embryoid body. Lab Chip 11, 874–882 (2011). 21249238 10.1039/c0lc00516a

[b26] Hattori K., Sugiura S. & Kanamori T. Microenvironment array chip for cell culture environment screening. Lab Chip 11, 212–214 (2010). 21076778 10.1039/c0lc00390e

[b27] Titmarsh D. M., Chen H., Glass N. R. & Cooper-White J. J. Concise Review: Microfluidic Technology Platforms: Poised to Accelerate Development and Translation of Stem Cell-Derived Therapies. Stem Cells Transl. Med. 3, 81–90 (2014). 24311699 10.5966/sctm.2013-0118 PMC3902292

[b28] Thorsen T., Maerkl S. J. & Quake S. R. Microfluidic Large-Scale Integration. Science 298, 580–584 (2002). 12351675 10.1126/science.1076996

[b29] Spurgeon S. L., Jones R. C. & Ramakrishnan R. High Throughput Gene Expression Measurement with Real Time PCR in a Microfluidic Dynamic Array. Plos One 3, e1662 (2008). 18301740 10.1371/journal.pone.0001662 PMC2244704

[b30] Lecault V. . High-throughput analysis of single hematopoietic stem cell proliferation in microfluidic cell culture arrays. Nat. Methods 8, 581–U593 (2011). 21602799 10.1038/nmeth.1614

[b31] Valero A. . Gene transfer and protein dynamics in stem cells uing single cell electroporation in a microfluidic device. Lab Chip 8, 62–67 (2008). 18094762 10.1039/b713420g

[b32] Kawada J., Kimura H., Akutsu H., Sakai Y. & Fujii T. Spatiotemporally controlled deliever of soluble factors for stem cell differentiation. Lab Chip 12, 4508–4515 (2012). 22968416 10.1039/c2lc40268h

[b33] Chung B. G. . Human neural stem cell growth and differentiation in a gradient-generating microfluidic device. Lab Chip 5, 401–406 (2005). 15791337 10.1039/b417651k

[b34] Sim W. Y. . A pneumatic micro cell chip for the differentiation of human mesenchymal stem cells under mechanical stimulation. Lab Chip 7, 1775–1782 (2007). 18030400 10.1039/b712361m

[b35] Zhang B., Green J. V., Murthy S. K. & Radisic M. Label-Free Enrichment of Functional Cardiomyocytes Using Microfluidic Deterministic Lateral Flow Displacement. Plos One 7, e37619 (2012). 22666372 10.1371/journal.pone.0037619 PMC3362623

[b36] Annabi N. . Hydrogel-coated microfluidic channels for cardiomyocyte culture. Lab Chip 13, 3569–3577 (2013). 23728018 10.1039/c3lc50252j PMC3744594

[b37] Boudou T. . A Microflabricated Platform to measure and Manipulate the Mechanics of Engineered Cardiac Microtissues. Tissue Eng. Part A 18, 910–919 (2012). 22092279 10.1089/ten.tea.2011.0341 PMC3338105

[b38] Myers F. B., Zarins C. K., Abilez O. J. & Lee L. P. Label-free electrophysiological cytometry for stem cell-derived cardiomyocyte clusters. Lab Chip 13, 220–228 (2013). 23207961 10.1039/c2lc40905d PMC3556464

[b39] Titmarsh D. M. . Microbioreactor Arrays for Full Factorial Screening of Exogenous and Paracrine Factors in Human Embryonic Stem Cell Differentiation. Plos One 7, e52405 (2012). 23300662 10.1371/journal.pone.0052405 PMC3530582

[b40] Titmarsh D. M., Ovchinnikov D. A., Wolvetang E. J. & Cooper-White J. J. Full factorial screening of human embryonic stem cell maintenance with multiplexed microbioreactor arrays. Biotechnol. J. 8, 822–834 (2013). 23813764 10.1002/biot.201200375

[b41] Frith J. E., Titmarsh D. M., Padmanabhan H. & Cooper-White J. J. Microbioreactor Array Screening of Wnt Modulators and Microenvironmental Factors in Osteogenic Differentiation of Mesenchymal Progenitor Cells. Plos One 8, e82931 (2013). 24376608 10.1371/journal.pone.0082931 PMC3871528

[b42] Ying Q.-L. . The ground state of embryonic stem cell self-renewal. Nature 453, 519–523 (2008). 18497825 10.1038/nature06968 PMC5328678

[b43] Sinha S. & Chen J. K. Purmorphamine activates the Hedgehog pathway by targeting Smoothened. Nat. Chem. Biol. 2, 29–30 (2006). 16408088 10.1038/nchembio753

[b44] Kamei K.-i. . Microfluidic image cytometry for quantitative single-cell profiling of human pluripotent stem cells in chemically defined conditions. Lab Chip 10, 1113–1119 (2010). 20390128 10.1039/b922884e PMC2970622

[b45] Flaim C. J., Teng D., Chien S. & Bhatia S. N. Combinatorial signaling microenvironments for studying stem cell fate. Stem Cells Dev. 17, 29–39 (2008). 18271698 10.1089/scd.2007.0085

[b46] Robertson C., Tran D. D. & George S. C. Concise Review: Maturation Phases of Human Pluripotent Stem Cell-Derived Cardiomyocytes. Stem Cells 31, 829–837 (2013). 23355363 10.1002/stem.1331 PMC3749929

[b47] Cheng H., Woodgett J., Maamari M. & Force T. Targeting GSK-3 family members in the heart: A very sharp double-edged sword. J. Mol. Cell. Cardiol. 51, 607–613 (2011). 21163265 10.1016/j.yjmcc.2010.11.020 PMC3075376

[b48] D’Uva G. . ERBB2 triggers mammalian heart regeneration by promoting cardiomyocyte dedifferentiation and proliferation. Nat. Cell Biol. 17, 627–638 (2015). 25848746 10.1038/ncb3149

[b49] Lian X. . Robust cardiomyocyte differentiation from human pluripotent stem cells via temporal modulation of canonical Wnt signaling. Proc. Natl. Acad. Sci. USA 109, E1848–E1857 (2012). 22645348 10.1073/pnas.1200250109 PMC3390875

[b50] Duffy D. C., McDonald J. C., Schueller O. J. A. & Whitesides G. M. Rapid Prototyping of Microfluidic Systems in Poly(dimethylsiloxane). Anal. Chem. 70, 4974–4984 (1998). 21644679 10.1021/ac980656z

[b51] Briggs J. A. . Integration-Free Induced Pluripotent Stem Cells Model Genetic and Neural Developmental Features of Down Syndrome Etiology. Stem Cells 31, 467–478 (2013). 23225669 10.1002/stem.1297

[b52] Hudson J., Titmarsh D., Hidalgo A., Wolvetang E. & Cooper-White J. Primitive cardiac cells from human embryonic stem cells. Stem Cells Dev. 21, 1513–1523 (2012). 21933026 10.1089/scd.2011.0254

[b53] Carpenter A. . CellProfiler: image analysis software for identifying and quantifying cell phenotypes. Genome Biol. 7, R100 (2006). 17076895 10.1186/gb-2006-7-10-r100 PMC1794559

[b54] Kamentsky L. . Improved structure, function and compatibility for CellProfiler: modular high-throughput image analysis software. Bioinformatics 27, 1179–1180 (2011). 21349861 10.1093/bioinformatics/btr095 PMC3072555

