# Supplementary material for: Induction of Human iPSC-Derived Cardiomyocyte Proliferation Revealed by Combinatorial Screening in High Density Microbioreactor Arrays
Source: Sci Rep. 2016 Apr 21;6:24637. doi: 10.1038/srep24637 (PMC4838928; doi:10.1038/srep24637)
Supplement: Supplementary Information [file srep24637-s1.pdf]

Induction of Human iPSC-Derived Cardiomyocyte Proliferation Revealed by Combinatorial Screening in High Density Microbioreactor Arrays

Supplementary Information

Drew M. Titmarsh[a,b,#], Nick R. Glass[a], Richard J. Mills[b], Alejandro Hidalgo[a], Ernst J. Wolvetang[a], Enzo R. Porrello[b], James E. Hudson[b], Justin J. Cooper-White[a,c,d,*]

[a]Australian Institute for Bioengineering & Nanotechnology, The University of Queensland, St. Lucia, QLD 4072, AUSTRALIA.

[b]School of Biomedical Sciences, The University of Queensland, St. Lucia, QLD 4072, AUSTRALIA.

[c]School of Chemical Engineering, The University of Queensland, St. Lucia, QLD 4072, AUSTRALIA.

[d]Biomedical Manufacturing, Manufacturing Flagship, CSIRO, Clayton, Victoria 3169, AUSTRALIA.

Correspondence should be addressed to:
Professor Justin J. Cooper-White
Australian Institute for Bioengineering & Nanotechnology
Building 75, Corner Cooper & College Roads
The University of Queensland
St. Lucia QLD 4072 AUSTRALIA
Phone: +61 7 334 63858
Fax: +61 7 334 63973
Email: j.cooperwhite@uq.edu.au

[#]Present address:
Institute of Medical Biology
Agency for Science, Technology and Research (A*STAR)
8A Biomedical Grove
#06-06 Immunos
SINGAPORE 138648
Email: drew.titmarsh@imb.a-star.edu.sg

Supplementary Table 1 - HDMA Physical Parameters

Parameter	Unit	HDMA	96-well Plate	24-well Plate
**Single Culture Chamber Dimensions**				
Diameter	mm	0.513	6.35	15.5
	m	5.13E-04	6.35E-03	1.55E-02
Culture Area	mm^2	0.207	31.7	188
	cm^2	2.07E-03	3.17E-01	1.88E+00
	m^2	2.07E-07	3.17E-05	1.88E-04
Nominal Culture Area Specified	mm^2	0.207	31.7	200
	cm^2	2.07E-03	3.17E-01	2.00E+00
	m^2	2.07E-07	3.17E-05	2.00E-04
Height	mm	0.1	2.5	2.5
	m	1.00E-04	2.52E-03	2.50E-03
Nominal Volume	uL	0.021	80	500
	mL	2.07E-05	0.08	0.50
	m^3	2.07E-11	8.00E-08	5.00E-07
Surface-Area-to-Volume (SAV) Ratio	mm^2/mm^3	10	0.40	0.40
Plate to Bioreactor Concentration Factor	-	25	1.0	1.0
Minimum Feature Size	mm	0.05		
Maximum Feature Aspect Ratio (height/width)	-	2		
**Cell Seeding**				
Nominal Cell Surface Density	$/cm^2$	4.00E+04	4.00E+04	4.00E+04
Nominal Cell Seeding Suspension Concentration	/mL	4.00E+06	1.59E+05	1.60E+05
**HDMA Single Column Dimensions**				
Interconnect Width	mm	0.08		
Rows		50		
Column Area (Wells only)	mm^2	10.3		
	cm^2	0.103		
Total Column Area incl. Interconnects	mm^2	10.6		
	cm^2	0.106		
Volume	uL	1.06		
**HDMA Entire Culture Array Dimensions**				
Columns		162		
Nominal Culture Array Area	mm^2	1709		
	cm^2	17.09		
Volume	uL	171		
Total Culture Area incl. Outlets				
**Nominal Flow Conditions**				
Area per column	cm^2	0.106		
Flow	$uL/h/cm^2$	1		
Channel Flow	uL/h	0.148		
	m^3/s	4.12E-14		
Total Flow	uL/h	24.000		
Number of Syringes	-	8		
Syringe Flow	uL/h	3.000		
**[a]Reynolds Number, Re**				
Fluid Density	kg/m^3	1000		
Average Velocity (chamber)	m/s	8.022E-07		
Average Velocity (interconnect)	m/s	4.115E-06		
Hydraulic Diameter (chamber)	m	1.67E-04		
Hydraulic Diameter (interconnect)	m	1.00E-04		

Fluid Viscosity	Pa.s	1.00E-03
Re (chamber)	-	1.34E-04
Re (interconnect)	-	4.12E-04
**[b]Peclet Number, Pé**		
Diffusivity (O2)	m^2/s	3.30E-09
Diffusivity (Glucose)	m^2/s	6.00E-10
Diffusivity (Albumin)	m^2/s	7.00E-11
Diffusivity (40kDa GF)	m^2/s	8E-11
Pe (chamber) O2	-	4.07E-02
Pe (chamber) (Glucose)	-	2.24E-01
Pe (chamber) (Albumin)	-	1.92E+00
Pe (chamber) (GF)	-	1.68E+00
**[c]Shear Stress, τ**		
Shear stress (chamber)	Pa	4.81E-05
Shear stress (interconnect)	Pa	9.88E-05
**Fluid Flow and Replacement**		
Mean Residence Time (Chamber)	h	0.140
Mean Residence Time (Column)	h	7.12
Space Velocity (Chamber)	/h	7.17
Space Velocity (Column)	/h	0.140

Notes

[a] Calculated as $\mathrm{Re} = \rho Q D_h / \mu A$, where density (ρ) = 1×10^3 kg/m^3; Q represents flowrate (m^3/s); D_h represents hydraulic diameter; viscosity (μ) = 10^{-3} Pa.s; and A represents cross-sectional area. Density and viscosity assumed as for water.

[b] Calculated as $\mathrm{Pe} = Q D_h / D_{AB} A$, where D_{AB} represents the diffusion coefficient.

[c] Shear stress estimated as $\tau = 6 \mu Q / h^2 w$, where height (h) = 2.5×10^{-4} m; and width (w) = 1.63×10^{-3} m for chamber at full width and 2.5×10^{-4} m for chamber interconnect.

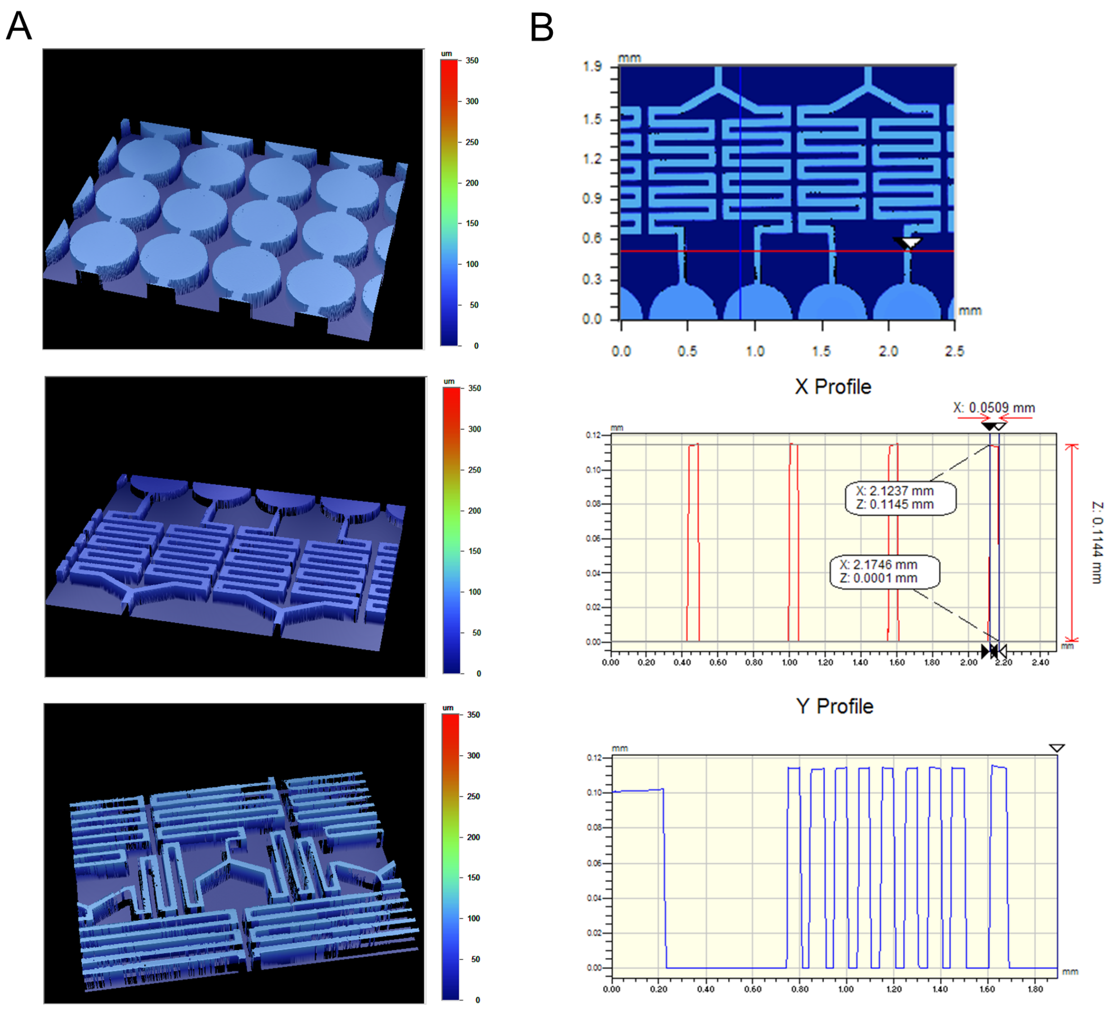

Supplementary Figure 1 - Optical Surface Profilometry. A 3D renderings of optical surface profiles of HDMA SU-8 masters. 2.5 mm x 1.9 mm sections of the cell culture array and fluidic channels are shown. **B** 2D representation and traced profiles showing height and width of microfabricated features (horizontal and vertical axes not in proportion).

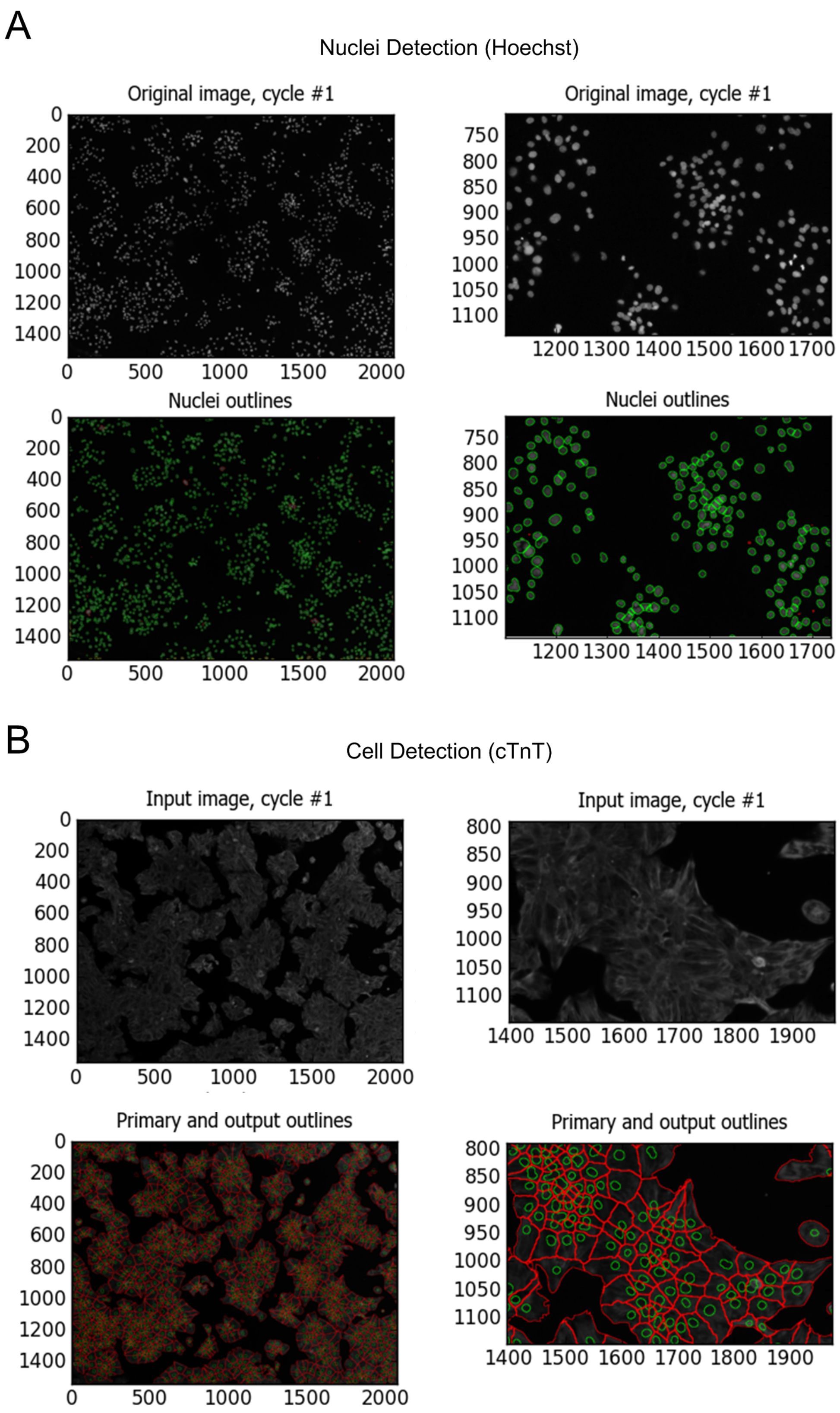

Supplementary Figure 2 – Image Cytometry Processing by CellProfiler. A CellProfiler input and output images for primary object (nuclei) identification, whole image and close-up, from static control experiments. **B** CellProfiler input and output images for secondary object (cell) identification, whole image and close-up, from static control experiments.

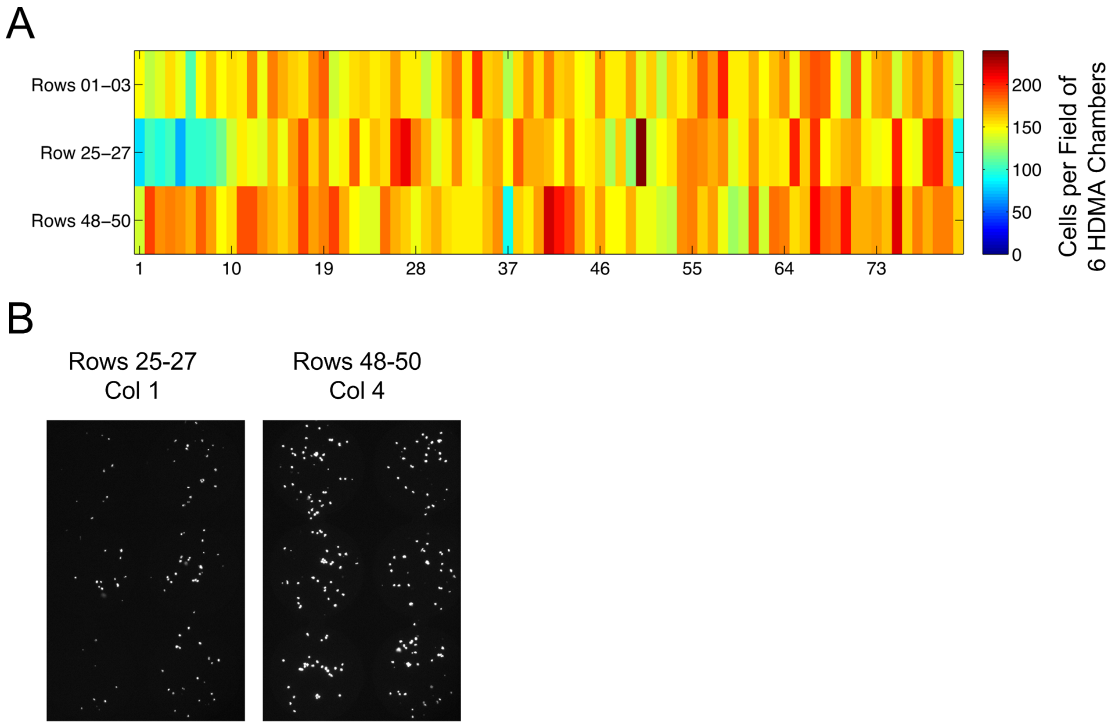

Supplementary Figure 3 - Cell Seeding Distribution. A Heatmap showing distribution of fixed, Hoechst-labelled HES3 hESCs after injection into HDMA at 2×10^6 cells/mL. Image fields containing 6 HDMA chambers (three rows of one column pair) were acquired and nuclei counted by image cytometry. Sets of three rows were taken from the top (Rows 01-03), middle (Rows 25-27), and bottom (Rows 48-50) were measured. **B** Example images of a less dense (left), and normal density (right) condition.

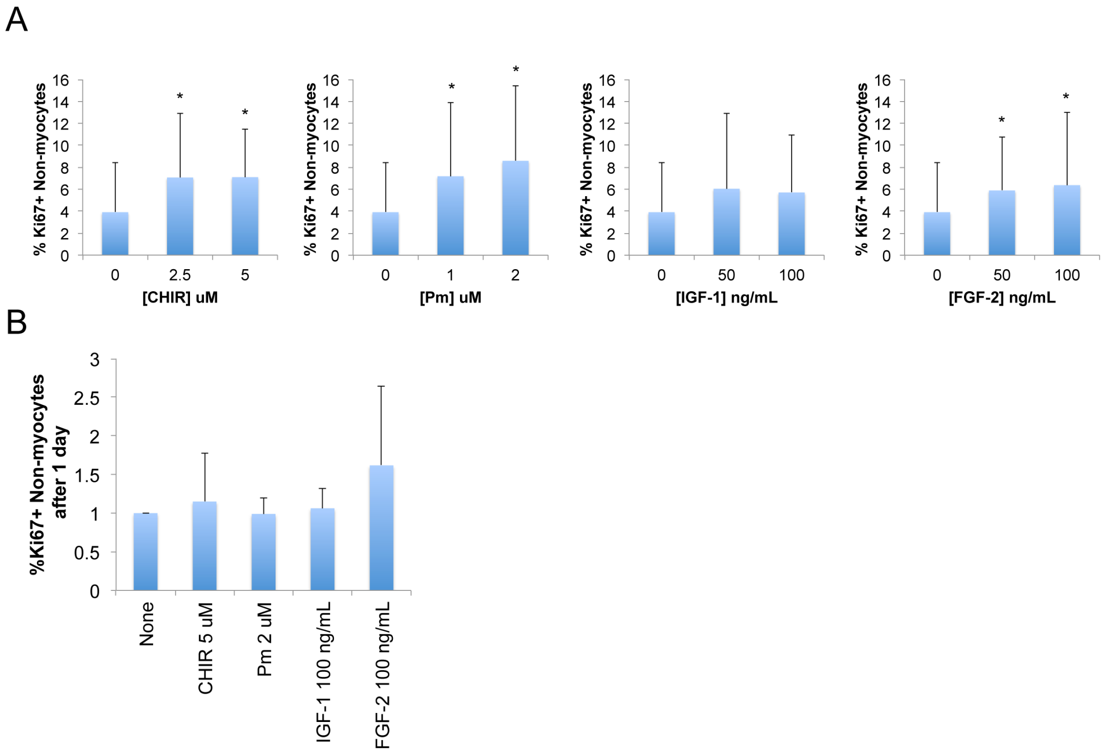

Supplemetary Figure 4 – Non-myocyte Proliferative Response in HDMA Screening. A Data from individual column-pairs representing treatment with individual factors in HDMA. Bars represent mean and error bars represent S.D. of 50 rows from each column-pair where corresponding chambers in the 2 replicate columns are averaged. * indicates $p < 0.05$ versus control (no factors) by unpaired two-tailed *t*-test. **B** Quantification of percentage cTnT$^-$Ki67$^+$ non-myocytes after 24 h treatment in static cultures. Bars represent mean +/- S.D. of 3 independent experiments including separate cardiomyocyte inductions, normalised to control (None) conditions. No treatments were significantly different (paired two-tailed *t*-test) against control conditions.

Supplementary Videos

Supplementary Video 1 – Assay startpoint, video of spontaneously contracting C32 iPSC-derived cardiomyocytes in HDMA.

Supplementary Video 2 - Assay endpoint (3 days), video of spontaneously contracting C32 iPSC-derived cardiomyocytes in HDMA.
